# The Effects of Natural Insecticides on the Green Peach Aphid *Myzus persicae* (Sulzer) and Its Natural Enemies *Propylea quatuordecimpunctata* (L.) and *Aphidius colemani* Viereck

**DOI:** 10.3390/insects15070556

**Published:** 2024-07-22

**Authors:** Francesco Lami, Giovanni Burgio, Serena Magagnoli, Laura Depalo, Alberto Lanzoni, Elettra Frassineti, Ilaria Marotti, Mattia Alpi, Dario Mercatante, Maria Teresa Rodriguez-Estrada, Giovanni Dinelli, Antonio Masetti

**Affiliations:** DISTAL-Department of Agricultural and Food Sciences, University of Bologna, 40127 Bologna, Italy; giovanni.burgio@unibo.it (G.B.); serena.magagnoli4@unibo.it (S.M.); laura.depalo@unibo.it (L.D.); alberto.lanzoni2@unibo.it (A.L.); elettra.frassineti2@unibo.it (E.F.); ilaria.marotti@unibo.it (I.M.); mattia.alpi3@unibo.it (M.A.); dario.mercatante2@unibo.it (D.M.); maria.rodriguez@unibo.it (M.T.R.-E.); giovanni.dinelli@unibo.it (G.D.); antonio.masetti@unibo.it (A.M.)

**Keywords:** essential oils, pyrethrins, insecticidal soaps, insecticide selectivity, parasitoid, Coccinellidae

## Abstract

**Simple Summary:**

Botanical insecticides and soaps are frequently considered environmentally friendly and compatible with the biological control of pests because of their purported higher selectivity towards beneficial insects when compared with synthetic insecticides. These claims are, however, only partially backed up by the scientific literature, thus justifying a case-by-case evaluation of botanical insecticides. Here, we tested the efficacy of five botanical insecticides and soaps belonging to different categories (pyrethrins, essential oils, crude plant extracts and insecticidal soaps) on the important aphid pest *Myzus persicae* (Sulzer) and their selectivity towards two of its biological control agents, the ladybird beetle *Propylea quatuordecimpunctata* (L.) and the parasitoid *Aphidius colemani* (Dalman). The pyrethrin-based product was the most effective against aphids but more detrimental to ladybird beetle larvae when compared to the alternatives. Some detrimental effects on ladybird beetles were also caused by white thyme essential oil, sweet orange essential oil and Marseille soap. Additionally, although pyrethrins have a broader spectrum of insecticidal activity compared to most botanical insecticides, they had no significant adverse effect on adult ladybird beetles and parasitoids within aphid mummies, similar to most of the other tested natural products.

**Abstract:**

Botanical insecticides and soaps are frequently proposed as environmentally safer alternatives to synthetic insecticides. However, the efficacy and selectivity of these products are often only partially supported by empirical evidence. Here, we tested the effectiveness of five botanical insecticides, belonging to different categories, on the green peach aphid *Myzus persicae* (Sulzer) and their selectivity towards two natural enemies, the ladybird beetle *Propylea quatuordecimpunctata* (L.) and the parasitoid *Aphidius colemani* (Dalman). White thyme essential oil (EO), sweet orange EO, crude garlic extract and Marseille soap were tested and compared with a pyrethrin-based commercial product. Both direct spray assays and residual contact assays on treated cabbage leaf disks were carried out. The tested products had low efficacy against aphids when compared to pyrethrins but were in general less detrimental to ladybird beetle larvae, meaning that if applied against other pests, they have a lower chance of harming this agent of aphid biocontrol. Some of the products (soap, orange EO) did, however, show direct exposure toxicity toward ladybird larvae, and thyme EO had extensive phytotoxic effects on cabbage leaves, possibly indirectly leading to higher mortality in ladybird adults. These results underline the necessity for case-by-case evaluations of botanical insecticides.

## 1. Introduction

Arthropod pests are the cause of massive crop losses worldwide [1,2], prompting the need for efficient defense strategies. Synthetic insecticides are amongst the most widely employed tools in this sense, and their use has steadily grown in recent decades [3]. These pesticides are, however, associated with a variety of negative effects, ranging from decreasing biodiversity [4] to the selection of insecticide-resistant strains in target pests [5]. Alternative approaches to managing pests while simultaneously limiting the need for pesticide applications are thus urgently needed.

Biological control is considered a key pillar of Integrated Pest Management (IPM) strategies [6,7] and it is hailed, in many cases, as an environmentally safe alternative to most synthetic pesticides. Conservative or augmentative strategies using selected biological control agents in agroecosystems can indeed achieve a high rate of pest suppression [8,9,10]. IPM frameworks thus place particular emphasis on the correct choice and mode of use of pesticides, as they can lead to negative effects on natural enemies [11,12], which, in turn, can end up limiting their potential to control pests (or even worsening pest-related problems) in the long term [13]. An example of these issues is represented by the emergence of previously low-damaging secondary pests once their natural enemies have been reduced by a pesticide aiming to control another pest species [14]. Ideally, highly selective pesticides should be employed in order to suppress pest populations while having little to no detrimental effects on beneficial organisms [15].

A steadily growing area of research in this respect is represented by botanical insecticides [16]. Some botanicals, such as pyrethrins and neem, have been widely employed against pests, especially in organic agriculture [17]. More recently, a wider array of products based on plant essential oils (EOs) have been introduced into the market [18], but they are still niche products in many sectors [19]. Other natural products traditionally employed in organic agriculture include insecticidal soaps [20] and crude plant extracts [21]. Natural insecticides are often seen as more biodiversity-friendly than synthetic alternatives [22], one of the reasons being that they usually have a much lower persistence in the environment [23]. Additionally, in cases in which they can be easily produced by using local plant species (for instance, as crude plant extracts), they can represent a cheaper alternative to commercial products [24], particularly in developing countries.

As the body of research concerning botanical insecticides grows, however, a more complex picture emerges, especially regarding their effectiveness and selectivity. While many papers report cases of successful applications of botanicals against pests and a degree of selectivity against non-target organisms [25,26,27,28], other studies have found that some botanical pesticides can negatively affect beneficial arthropods [29,30,31] or are scarcely effective against pests [32,33]. Moreover, studies focusing on botanical insecticides often have limited reproducibility due to flaws in their experimental design [34]. Because of their low environmental persistence, even botanical insecticides that have been shown to be effective in laboratory assays may have low efficacy against pests in field conditions [35]. It is thus clear that the evaluation of novel botanical insecticides cannot be based on broad generalizations [36] but should proceed on a case-by-case basis. This is especially important in the context of organic farming, in which synthetic pesticides are banned [37,38] and thus pest control relies heavily on alternative methods, including natural products, biological control [39] and their potential integration.

The aim of this study was to test the effects of botanical insecticides and soaps representing different categories (pyrethrins, EOs, insecticidal soaps and crude plant extracts) on an important insect pest and its natural enemies, both through direct spray applications and residue exposure assays. The green peach aphid *Myzus persicae* (Sulzer) (Hemiptera: Aphididae) was chosen, as it is a widely distributed species that causes severe damage to several crops in temperate areas [40,41,42], both by direct feeding activity and by vectoring plant pathogens [43]. As for natural enemies, the predatory 14-spot ladybird beetle *Propylea quatuordecimpunctata* (L.) (Coleoptera: Coccinellidae) and the aphid parasitoid *Aphidius colemani* (Dalman) (Hymenoptera: Braconidae) were tested. Both species are efficient aphid biocontrol agents that are widespread in Europe [44,45,46,47], where they are frequently used in augmentative biological control programs.

## 2. Materials and Methods

### 2.1. Insect Rearing

Green peach aphids were reared on green pea sprouts (*Pisum sativum* L.) in the laboratories of the Department of Agricultural and Food Sciences of the University of Bologna [48]. The rearing temperature was 20 °C, with 70% relative humidity and a 16:8 photoperiod.

*Propylea quatuordecimpunctata* adults and egg masses and mixed-age aphid mummies containing *A. colemani* were provided by Bioplanet srl (Cesena, Italy). The ladybird eggs were maintained at 25 °C, with 75% relative humidity and a 16:8 photoperiod, until hatching. Larvae were held under the same conditions and fed ad libitum with green peach aphids and frozen mill moth eggs (*Ephestia kuehniella* Zeller) until molting to the second instar, which were then used in the assays. The mill moth eggs were also provided by Bioplanet srl.

In the case of the parasitoids, spray treatments were tested on mummies rather than adult parasitoids because the small size and fragility of this species were likely to make it susceptible to physical damage from spraying with the employed spray bottle, as verified through some preliminary trials using water. Consequently, part of the parasitoid mummies was left to hatch at 25 °C, with 70% relative humidity and a 16:8 photoperiod, in order to obtain the adults needed for the residue exposure assays, while the rest was immediately used for the spray treatment experiment. Adult parasitoids were fed on a 50% honey solution dripped directly onto the filter paper used as a lid for the cups containing the insects.

The same temperature, relative humidity and photoperiod parameters reported here were also applied to the insects subjected to the trials.

### 2.2. Insecticides

Five insecticides of natural origin were tested: white thyme (*Thymus vulgaris* L.) EO (provided by GreenVet srl, Forlì, Italy), Prev-Am^®^ Plus (a formulated product containing 60 g/L of sweet orange Citrus × sinensis (L.) Osbeck EO, produced by Oro Agri Europe S.A., Palmela, Portugal), crude garlic extracts (*Allium sativum* L.), Marseille soap (“Sapone di Marsiglia per bucato in filetti” EcorNaturaSì^®^, Sand Vendemiano, Italy) as an insecticidal soap [16] and Rabona^®^ (a product based on 5% pure pyrethrins) provided by Serbios srl, Badia Polesine, Italy (Table 1). The latter was used as a reference commercial product given the known broad spectrum of pyrethrins [49]. These compounds are representative of a variety of different insecticide types and were selected from a wider insecticide array after a preliminary trial on green peach aphids (Supplementary Materials Chapter S1, Tables S1 and S2, Figures S1–S3). Distilled water was used to dilute the products and also as the negative control.

The products based on orange EO and pyrethrins were used at the recommended concentrations for aphids in open field conditions (0.5% and 0.064%, respectively). Marseille soap was used at a concentration of 5 g/L (0.5%), comparable with the LC50 concentration for adult green peach aphids using a commercial insecticidal soap [50]. White thyme EO was used at a concentration of 0.25%, which was considered economically sustainable for the field application of EOs; this EO was diluted in water using a plant-based emulsifying agent as a solubilizer (0.5%), which was provided by Vulcaplant srl (Valsamoggia, Italy). The crude garlic extract was obtained by leaving commercial garlic powder for organic agriculture (Cerrus^®^, Uboldo, Italy) in distilled water (8 g/L) for 10 days at room temperature (24 ± 2 °C). Marseille soap (0.5%) was added to the resulting extract before use, as preliminary trials showed that this combination was more effective on green peach aphids than the extract alone (Supplementary Materials Chapter S1). For brevity, the mix of crude garlic extract and Marseille soap will hereafter be referred to as simply the “crude garlic extract”, unless otherwise stated.

### 2.3. Chemical Analyses

Studies on botanical insecticides often lack chemical characterization of the tested products [34]. This is a significant weakness, as one given plant species can have many chemotypes, also differing in insecticidal activity [51]. In this study, therefore, Marseille soap, crude garlic extract, white thyme EO and the solubilizer used with the latter were chemically characterized. The orange EO and pyrethrins, on the other hand, were not characterized because the concentrations of the active ingredients are readily available on the labels of the respective commercial products.

Volatile organic compound (VOC) analysis of the four products was performed by SPME-GC-MS (Shimadzu, Kyoto, Japan) according to [52], with some modifications. For the Marseille soap, its fatty acid composition was also characterized. Lipids were first extracted according to a modified version of the Folch method [53] and then subjected to double methylation in a methanolic medium (first with sodium methoxide and then with boron trifluoride) to ensure that all the fatty acids (including the free ones) were completely methylated [54]. The fatty acid methyl esters were then injected into a GC-FID (Shimadzu, Kyoto, Japan) and quantified [55]. Additional details about the chemical procedures are reported in Supplementary Material Chapter S2.

### 2.4. Spray Application Assays

For the green peach aphids, plastic cups (diameter 6 cm, height 3.5 cm), each with a 0.5 cm layer of 2% agar gel, were prepared. A leaf disk (≈6 cm diameter) of Chinese cabbage (*Brassica rapa* L.) was laid on the agar with its abaxial surface facing up. Twenty adult green peach aphids were gently transferred onto the leaf disk in each cup and then sprayed with the insecticide (approximately 1 mL) until run-off using a household spray bottle, set at the finest available droplet size. A total of 6 replicate cups per treatment (including the water control) were prepared, and the experiment was repeated once, comprising a total of 72 cups (1440 aphids). Aphid mortality was checked 24 h after treatment; aphids unable to right themselves once turned on their back were considered dead.

The mortality of ladybird adults and parasitoids within mummies was tested using the same number of replicates of aphids (6 per treatment, repeated once—72 cups in total). Due to their irregular availability, the second instar ladybird larvae had a slightly higher number of replicates for this trial (1 extra replicate per treatment). Each replicate consisted of a plastic cup (diameter 6 cm, height 7.5 cm) housing 5 insects or mummies. Each group of 5 individuals was placed into an empty cup, sprayed with approximately 1 mL of treatment solution and then immediately transferred into a second plastic cup for the rest of the test. Ladybird adults and larvae were fed ad libitum with green peach aphids and *E. kuehniella* frozen eggs. As ladybirds are significantly larger, more sclerotized and longer-lived than aphids, their mortality was checked 72 h after treatment for both adults and larvae. The number of larvae that reached the adult stage within 10 days after treatment was also recorded. For parasitoids, the number of wasps that did not manage to successfully emerge from the mummies within 10 days was recorded as a measure of mortality.

### 2.5. Residue Exposure Assays

The official testing protocol (test method 019) of the Insecticide Resistance Action Committee (IRAC) (https://irac-online.org/pests/myzus-persicae/; accessed on 25 June 2024) was adopted to test the effects of the insecticide residues on green peach aphids. The protocol was similar to that described above for spray application, but before being placed on agarose gel, the leaf disks were dipped in the treatment solution for 10 s and then left to dry under a fume hood. Twenty adult aphids were then transferred onto the leaf surface, and their mortality was checked 24 h after aphid placement. A total of 6 replicates were performed for each treatment (including the water control), and the experiment was repeated once (72 cups in total).

The same protocol was applied to the natural enemies, with the only differences being the size of the plastic cups (diameter 6 cm, height 7.5 cm) and the inclusion of 5 insects in each cup. Due to their irregular availability, second instar ladybird larvae had a slightly lower number of replicates for this trial (1 less replicate for thyme EO, orange EO, soap and pyrethrins). Ladybird adults and second instar larvae were fed ad libitum on green peach aphids and *E. kuehniella* frozen eggs. The mortality was checked after 72 h for both adults and larvae. The number of larvae that were able to reach the adult stage within 10 days after treatment was also recorded.

Adult parasitoids were fed on a 50% honey solution dripped onto the filter paper used as a lid for the cups. Their mortality was checked 24 h after placing the wasps in the cups. Because of the high mortality (42.1%) recorded in the negative control, the experimental setup was considered unsuitable for gaining meaningful information, and the resulting data were not included in the statistical analyses.

During the residue exposure assays, an evident phytotoxic effect with extensive leaf tissue damage was frequently observed by visual inspection in the leaf disks treated with white thyme EO. Therefore, the leaf disks were ranked into two classes on the basis of the extensions of the necrotic areas: (i) less than 25% of leaf surface and (ii) more than 25% of leaf surface.

### 2.6. Statistical Analysis

Insecticidal efficacy was calculated according to the Henderson–Tilton formula [56] for each insecticide treatment and insect.

For the aphids, adult ladybird beetles and ladybird larvae, two binomial Generalized Linear Models (GLMs) with a logit link function were calculated, one testing the effect of direct spray application and the other testing the effects of contact with the residues (Models 1–6). As previously stated, the dependent variable was represented by the mortality 24 h after treatment/exposure to the residues for aphids and by the mortality 72 h after treatment/exposure to residues both for adult and larval ladybird beetles. Similar GLMs were calculated to test the effects of the spray and residue treatments on the number of ladybird larvae able to reach the adult stage; the results were, however, very similar to the results of the models for larval mortality after 72 h, which is not surprising given the high correlation between these two parameters both for the spray treatment (Pearson’s r = −0.94; *p* < 0.001) and the exposure to residues (Pearson’s r: −0.73; *p* < 0.001). Therefore, only the results of the larval mortality models were included here. A similar GLM (Model 7) was calculated for parasitoids, testing the effect of the spray treatments on the number of individuals that were unable to emerge from the aphid mummies within 10 days after the treatment was performed. In the case of omnibus significant differences being detected by the GLMs, post hoc tests with Tukey’s adjustment were carried out to compare the different treatments.

All the statistical analyses were performed with R v3.6.2 [57] using the base package stats, as well as the package “emmeans” v1.4.4 [58] for multiple comparisons.

## 3. Results

### 3.1. Chemical Composition and Phytotoxic Effects

Regarding the VOC composition, sulfur compounds were the most relevant class (~67% of the total detected compounds) in the crude garlic extracts (Table 2), followed by acids, terpenes and alcohols. In the samples of white thyme EO, terpenes were the most represented class (~71% of total compounds, with ~28% being thymol), followed by alkenes, acids and alcohols (Table 2). In the solubilizer, acids were the most relevant compounds (~82% of total compounds), followed by alkenes, ketones, terpenes, aldehydes and alcohols (Table 2). In the mixture of thyme EO and the solubilizer (0.25:0.5% in water), its chemical composition reflects the VOC composition of the single blended components and their ratio, as terpenes were the most abundant (~71% of total compounds), followed by alkanes and acids (Table 2). In the Marseille soap samples, alkenes were the most abundant VOCs (~76%), followed by aldehydes, ketones, terpenes, alcohols and acids (Table 2). Detailed VOC compositions of the tested products are reported in Tables S3–S7.

Regarding the Marseille soap’s fatty acid composition, the most abundant fatty acid was oleic acid (52.7% of total fatty acids), followed by palmitic acid (18.8%) and linoleic acid (12.6%) (Table S8).

As for the observed phytotoxic effects, 23 out of 35 leaf disks treated with white thyme EO during the residue exposure trials showed necrotic areas covering more than 25% of their surface, while 10 out of 35 leaf disks showed damage on less than 25% of their surface, and only 2 leaf disks were completely undamaged.

### 3.2. Insect Mortality

**Figure 1:**
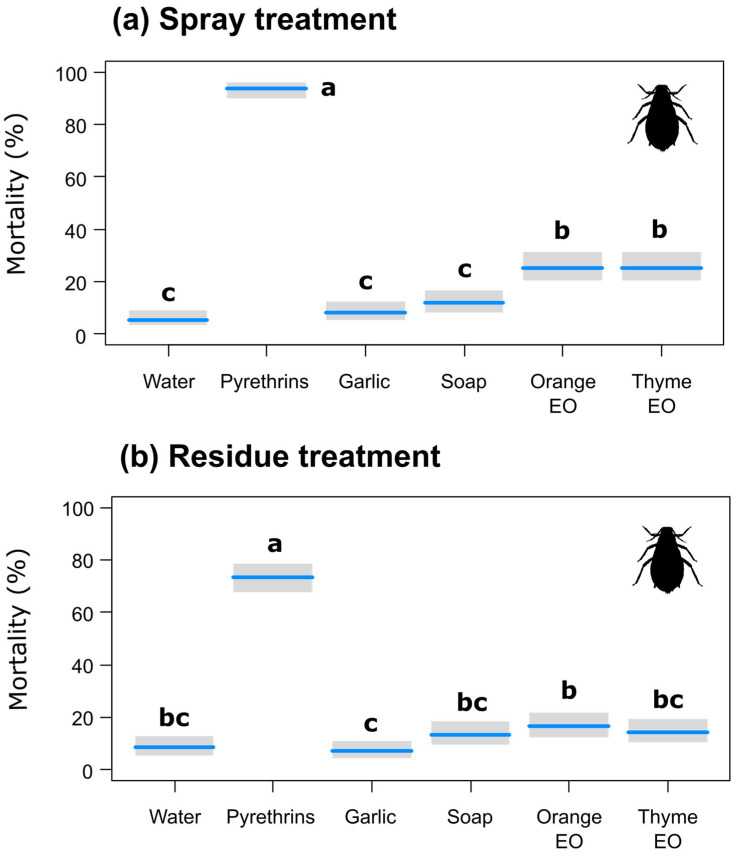
Effects of the botanical insecticides on green peach aphid adults in the spray application (**a**) and exposure to residues (**b**) bioassays, with gray bands representing Wald-type confidence intervals. Different letters indicate statistically significant differences as detected by post hoc pairwise comparisons with Tukey’s adjustment (*p* < 0.05). EO = essential oil.

The mean mortality was relatively low with high variability in most bioassays. In the spray application assays, pyrethrins were the most effective insecticide against green peach aphids (Henderson–Tilton-corrected efficacy of 93.4%), causing a significantly higher mortality than all the other treatments (Table 3). The mortality caused by spray applications of the orange and thyme EOs (corrected efficacy of 21.0% for both) was significantly higher than that caused by the negative control but lower than that caused by pyrethrins (Table 3, Figure 1a).

In the residue exposure assays, only pyrethrins caused a significantly higher aphid mortality than water (Table 3, Figure 1b; corrected efficacy of 71.1%). Orange EO showed a higher insecticidal activity than garlic extract, but none of the products significantly differed from the negative control.

**Figure 2:**
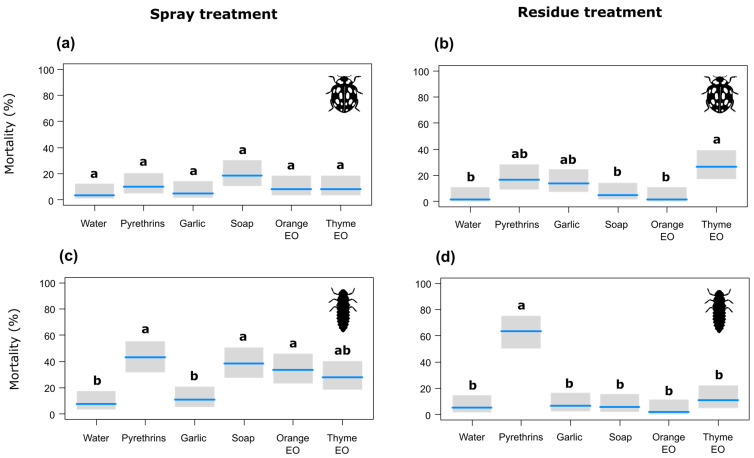
Effects of the botanical insecticides on adult 14-spot ladybird beetles in spray (**a**) and residue exposure (**b**) assays and on ladybird larvae in spray (**c**) and residue exposure (**d**) assays, with gray bands representing Wald-type confidence intervals. Different letters indicate statistically significant differences according to the post hoc pairwise comparison test with Tukey’s adjustment (*p* < 0.05). EO = essential oil.

In the spray application assays on the adult ladybird beetles, no significant differences in mortality were detected among treatments (Table 3, Figure 2a). Similar results were found in the residue exposure assays, except for white thyme EO, which was the only product causing a significantly higher mortality than water (corrected efficacy of 25.4%), Marseille soap and orange EO (Figure 2b).

In the spray application assays on the ladybird larvae, pyrethrins (corrected efficacy of 38.3%), orange EO (corrected efficacy of 28.2%) and Marseille soap (corrected efficacy of 33.3%) caused a significantly higher mortality than the water control and garlic extract (Figure 2c). Only pyrethrins (corrected efficacy of 61.7%) caused a significantly higher mortality than water and all the other treatments in the residue exposure assays (Figure 2d).

**Figure 3:**
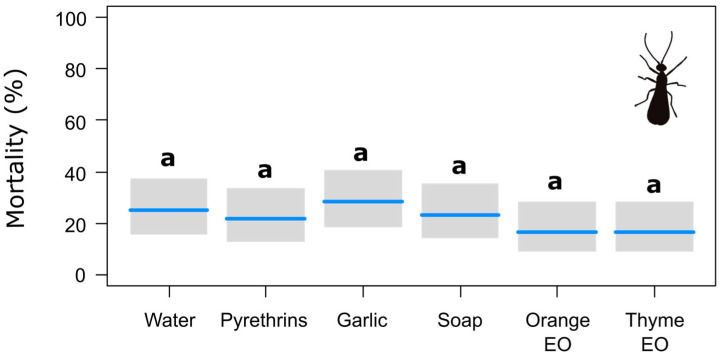
Effects of the botanical insecticides on the emergence of adult parasitoids from the sprayed aphid mummies, with gray bands representing Wald-type confidence intervals. The same letter over each band indicates no statistically significant differences according to the post hoc pairwise comparison test with Tukey’s adjustment (*p* < 0.05). EO = essential oil.

Finally, there were no significant differences among treatments in terms of the number of parasitoids failing to emerge from the mummies within 10 days after the spray treatment (Table 3, Figure 3). It is worth noting that considering failure to emerge to be equivalent to death, the parasitoid trials were those with the highest mortality with the water control, exceeding 25%. Nevertheless, this is within the range of normal mortality for *A. colemani* reared on *M. persicae* at 25 °C [59].

## 4. Discussion

The present research shed some light on the effectiveness and selectivity of different botanical insecticides in the context of green peach aphid control. First of all, the results of this study clearly highlighted that pyrethrins had a higher insecticidal activity against green peach aphids than the other tested products. This is in line with several studies reporting the important insecticidal activity of pyrethrum extracts [49,60].

The products based on white thyme and orange EOs showed partial efficacy against aphids. The tested orange EO product is marketed as an insecticide targeting soft-bodied arthropods (including aphids) and, more generally, the literature reports several cases of EOs, including white thyme EO, acting as effective insecticides against various aphid species, including green peach aphids [61,62,63]. Thymol, the most abundant compound in thyme EO, has been found, for instance, to be effective against *Pochazia shantungensis* Chou & Lu (Hemiptera: Ricaniidae) when using the leaf-dipping method [64]. Differences in the reported efficacy levels might be related to differences in EO type, concentration and exposure mode, which may vary widely among studies. Also, the chemical composition of EOs, which is known to be affected by several factors, such as geographical origin, pedo-climatic conditions, harvest time, the plant part used and extraction technique, can influence the insecticidal activity [34].

Although the botanical insecticides and soaps investigated in this study showed limited potential (in the case of the EOs) or even no effect (in the case of the soap and garlic extract) on adult green peach aphids, the tested products showed a much higher efficacy against aphid nymphs in the preliminary trials (Figures S1–S3), potential that deserves to be investigated further. Additionally, these products continue to be studied in relation to other pest insects and plant pathogens, with some promising results on a number of dipteran, homopteran and lepidopteran pests [65,66,67,68,69], as well as fungal pathogens [70,71]. For such products, which may see real-world applications in field conditions, an overall picture of their potential non-target effects would be important. Aside from the natural enemies of the target pest, these assessments should focus on a range of biological control agents. Highly selective insecticides, which promote the conservation of aphid natural enemies, would reduce the chances of aphids emerging as secondary pests [14] and in general would preserve pre-existing communities of beneficial organisms that could spread to nearby crops and provide useful ecosystem services, such as biological control [72,73].

In this respect, when tested on adult 14-spot ladybird beetles, thyme EO, orange EO, garlic extract and Marseille soap had no significant effects, but the same was also true for pyrethrins. Although pyrethrins are considered broad-spectrum insecticides [49], in both the spray and residue exposure assays their effects on adult ladybird beetles did not statistically differ from the water control. A low susceptibility of ladybirds to pyrethrins has also been reported for *Coccinella septempunctata* L. and *Harmonia axyridis* Pallas [74,75]. A lack of insecticidal activity on adult 14-spot ladybird beetles was also observed for the other tested natural products, except for white thyme EO, which caused a significantly higher mortality than the water control but only in the residue exposure assays. Phytotoxic effects on leaf disks treated with white thyme were frequently observed in these trials; specifically, all the leaf disks used for the residue exposure assays of the adult ladybirds showed some symptoms of phytotoxicity, and in 75% of cases, over half of the leaf surface was damaged. It thus cannot be excluded that the VOCs released from the damaged leaves were partially responsible for the observed ladybird mortality due to biofumigation [76,77]. This would explain why thyme caused no significant mortality in the spray application assays, where treated leaf disks were not used.

The situation was remarkably different for ladybird beetle larvae. In the spray application assays, the Marseille soap and orange EO caused a level of mortality similar to pyrethrins and significantly higher mortality than the water control, while the white thyme EO and garlic extract proved to be more selective and did not cause a significantly higher mortality than water. On the other hand, only pyrethrins caused a significantly higher mortality than water during the residue exposure trials. The different susceptibility of adult and larvae ladybird beetles to pyrethrins might be explained by differences in their integument permeability and in their levels of the pyrethrin-metabolizing enzyme glutathione transferase, which have been reported for other coccinellids [78,79].

None of the tested insecticides significantly altered the rate of emergence of the parasitoid *A. colemani* from the aphid mummies in the spray application assays. It is likely that the mummy offers some degree of protection to the insect from external toxicants, as also noted for other parasitoid species, including *Aphelinus semiflavus* Howard and *Diaretiella rapae* (McIntosh) [80,81]. The effect of spray or residue exposure of these products on *A. colemani* adults is, however, still unknown and should be investigated in future studies.

Overall, it can be inferred that aside from pyrethrins, the tested botanical insecticides would have little impact on green peach aphids in a real-world scenario. However, these products showed some differences in terms of their selectivity against biological control agents, which should be taken into account when considering their application to other pests or plant pathogens. Specifically, the tested products proved in most cases to be less impactful than pyrethrins on 14-spot ladybird beetles, as they do not harm the larvae of this species as residues and, in some cases (garlic extract and thyme EO), have negligible impacts on larvae even in spray treatment. The partial, although low, effectiveness of thyme and orange EOs on the tested aphids could thus be compensated for by their negligible impact on beneficial fauna, making their use possible in integrated strategies including biological control agents. However, negative effects on the natural enemies were not completely absent, as spray applications of Marseille soap and orange EO increased the mortality of the ladybird larvae and residues of white thyme EO were detrimental to ladybird adults, possibly indirectly through their phytotoxic effect on the cabbage leaves. While we did not investigate which specific component of the thyme EO (or the solubilizer) caused phytotoxicity, such effects, which have also been reported in other studies involving thyme EO [82], are likely caused by thymol [83] and are another element to consider before field applications. The pervasive damage observed in this study could be attributed to the extensive penetration of EO into leaf tissues through the cut margins of the leaf disks [84], and the use of this EO on living crop plants thus might not cause the same effects [85]. However, phytotoxicity is considered one of the main constraints when developing pesticides based on EOs [86] and, consequently, a careful evaluation on the main target crops in field conditions is highly recommended.

In conclusion, our study improved the knowledge of the potential and limitations of botanical insecticides and soaps in the context of green peach aphid control, also considering their non-target effects on important natural enemies. One of these enemies, the ladybird *P. quatuordecimpunctata*, was previously untested for its susceptibility to natural insecticides in spite of it being a commonly employed biocontrol agent in Europe, and thus our data are especially valuable in expanding the knowledge on the interactions between natural products and ladybird beetles. Our results confirmed the necessity of case-by-case evaluations of these products while avoiding broad generalizations; such evaluations could be carried out through laboratory trials on target and non-target organisms, followed by field testing of the most promising products. This is especially important given that botanical insecticide performance can be very heterogeneous when considering different aspects, such as its efficacy towards the target pest, selectivity towards natural enemies and effects on crop plants.

## Figures and Tables

**Table 1:** Tested insecticidal products.

Commercial Name	Active Ingredient	Tested Concentration (g/L)
Prev-Am^®^ Plus	Orange EO (60 g/L)	5
NA (provided by GreenVet srl),	White thyme EO	2.5
Cerrus^®^, Aglio	Crude garlic extract	8
EcorNaturaSì^®^	Marseille soap	5
Rabona^®^	Pyrethrins (50 g/L)	0.64

EO = essential oil.

**Table 2:** Volatile organic compound classes detected in analyzed samples. Detailed VOC compositions of the tested products are reported in Tables S3–S7. Data are expressed as the percentage of each VOC class in the total identified VOCs.

	VOC Classes (Internal Distribution, %)							
Samples	Alkenes	Aldehydes	Ketones	Sulfur Compounds	Terpenes	Alcohols	Acids	Others
Crude garlic extract (alone)	n.d.	n.d.	n.d.	67.81	10.89	3.14	15.65	2.52
Thyme EO	27.80	n.d.	n.d.	n.d.	71.87	0.16	0.17	n.d.
Plant-based solubilizer	12.71	0.99	2.69	n.d.	1.32	0.37	81.94	n.d.
Thyme EO + Plant-based solubilizer	27.74	n.d.	n.d.	n.d.	71.12	n.d.	1.14	n.d.
Marseille soap	76.00	12.32	2.58	n.d.	2.91	2.89	3.31	n.d.

EO = essential oil; n.d. = not detected.

**Table 3:** Results of the binomial Generalized Linear Models testing the effects of spray and residue exposure of treatments on the studied insects. In the case of parasitoids, mortality is given as the number of parasitoids that failed to emerge from mummies in the 10 days following the treatment.

Insects	Dependent Variable	Fixed Effect	d.f.	χ^2^	*p*
*Model 1*					
Aphids	Mortality at 24 h	Spray treatment	5	647.79	<0.001
*Model 2*					
Aphids	Mortality after 24 h	Residue treatment	5	381.95	<0.001
*Model 3*					
Adult ladybird beetles	Mortality at 72 h	Spray treatment	5	9.59	0.09
*Model 4*					
Adult ladybird beetles	Mortality after 72 h	Residue treatment	5	32.09	<0.001
*Model 5*					
Ladybird larvae	Mortality at 72 h	Spray treatment	5	38.85	<0.001
*Model 6*					
Ladybird larvae	Mortality after 72 h	Residue treatment	5	94.23	<0.001
*Model 7*					
Parasitoids	Mortality at 10 d	Spray treatment	5	3.81	0.58

## Data Availability

The raw data supporting the conclusions of this article will be made available by the authors on request.

## Supplementary Materials

The following supporting information can be downloaded at https://www.mdpi.com/article/10.3390/insects15070556/s1: Chapter S1 (Preliminary screening of essential oils and crude plant extracts against the green peach aphid *Myzus persicae* (Sulzer)), including Figure S1: Effects of the different combinations of EOs and solubilizers on aphid mortality. Different letters indicate statistically significant differences according to Gabriel’s test; Figure S2: Effects of the different combinations of EOs and solubilizers on aphid mortality, including the commercial product Prev-Am^®^. One-way ANOVA: F (9, 127) = 5.05; *p* < 0.001. Different letters indicate statistically significant differences according to Gabriel’s test; Figure S3: Effects of the different combinations of crude plant extracts and Marseille soap on aphid mortality. Different letters indicate statistically significant differences according to Gabriel’s test; Table S1: Results of the factorial ANOVA testing the effect of EOs, solubilizers and their interaction on aphid mortality; Table S2: Results of the factorial ANOVA testing the effect of crude plant extracts, Marseille soap and their interaction on aphid mortality. The Supplementary Materials also include Chapter S2 (Chemical analysis), including Table S3: VOCs detected in crude garlic extract samples; Table S4: VOCs detected in white thyme EO samples; Table S5: VOCs detected in solubilizer samples; Table S6: VOCs detected in mixed samples of thyme EO (0.25%) and solubilizer (0.5%) in water; Table S7: VOCs detected in Marseille soap samples; Table S8: Fatty acid and fatty acid classes (expressed as % of total fatty acids) of Marseille soap [52,53,54,55,87].
