# Supplementary material for: The Effects of Natural Insecticides on the Green Peach Aphid Myzus persicae (Sulzer) and Its Natural Enemies Propylea quatuordecimpunctata (L.) and Aphidius colemani Viereck
Source: Insects. 2024 Jul 22;15(7):556. doi: 10.3390/insects15070556 (PMC11277335; doi:10.3390/insects15070556)
Supplement: Supplementary file 1 [file insects-15-00556-s001.zip › insects-3099710-supplementary.pdf]

Supplementary Materials

The Effects of Natural Insecticides on the Green Peach Aphid *Myzus persicae* (Sulzer) and Its Natural Enemies *Propylea quatuordecimpunctata* (L.) and *Aphidius colemani* Viereck

Francesco Lami, Giovanni Burgio, Serena Magagnoli, Laura Depalo, Alberto Lanzoni, Elettra Frassineti, Ilaria Marotti, Mattia Alpi, Dario Mercatante, Maria Teresa Rodriguez-Estrada, Giovanni Dinelli, Antonio Masetti

DISTAL-Department of Agricultural and Food Sciences, University of Bologna, 40127 Bologna, Italy.

Keywords: essential oils; pyrethrins; insecticidal soaps; insecticide selectivity; parasitoids; Coccinellidae

1. Preliminary screening of essential oils and crude plant extracts against the green peach aphid *Myzus persicae* (Sulzer)

Laboratory assays were carried out on the green peach aphid *Myzus persicae* (Sulzer) to select the most effective botanical insecticides based on essential oils and crude plant extracts to be included in the main experiments, as described in the paper.
The tested essential oils were white thyme *Thymus vulgaris* L., weeping paperbark *Melaleuca leucadendra* (L.) L. and clove *Syzygium aromaticum* ((L.) Merr. & L. M. Perry), using either a plant-based solubilizer (composition in Table S5) or micronized zeolite as solubilizers. All essential oils were tested at 0.25% (v/v) and solubilizer concentrations were 0.5%, as recommended by manufacturers. There was a total of six tested combinations (3 essential oils * 2 solubilizers), plus the controls (either the plant-based product or zeolite at 0.5% in distilled water) and the commercial product Prev-Am® (Oro Agri Europe S.A., Palmela, Portugal), based on sweet orange *Citrus sinensis* (L.) essential oil.
The tested crude plant extracts were garlic *Allium sativum* L., common nettle *Urtica dioica* L., chili pepper *Capsicum annuum* L., tomato *Solanum lycopersicum* L. and *Sedum* L. To obtain the extracts, garlic plant powder and fresh green materials for the remaining plants were left in distilled water at 8 g/L concentration and held at room temperature (24±2 ºC) for 10 days. All crude extracts were tested alone and mixed with 0.5% (5 g/L) Marseille soap. The soap was also tested alone at 0.5% and a negative water control was included in the assays.

1.1.Methods

Approximately 3-cm high green pea sprouts (*Pisum sativum* L.) were grown on agri-perlite. Two parthenogenic aphid females were placed on each sprout. After 48 hours, the adults were removed and aphid nymphs were counted. Only plants with 5 to 20 aphid nymphs were then used in the trials, leading to a variable number of replicates per treatment (11-17 replicates for essential oil treatments and 6-9 replicates for extract treatments). Infested sprouts were treated using a pump spray nebulizer until dripping, and aphid mortality was checked after 48 hours. Aphids unable to right themselves when turned on their backs were considered dead.

1.1.1. Data analysis

For EOs experiments percent mortalities recorded for each pea sprout were arcsine-transformed to meet the assumptions of normality and homoscedasticity and analyzed by factorial ANOVA considering EOs and solubilizer as interacting factors. In order to in include in the analysis also the commercial product Prev-Am®, which do not need a solubilizer to mix with water, a one-way ANOVA was also run on EOs considering as separate levels of the treatment factor each combination of EOs and solubilizer.
Mortality data recorded in the assays on plant extract were analyzed by a factorial ANOVA model considering the extracts and the soap as interacting factors. Gabriel's test, which is recommended as post-hoc method for samples of unequal sizes [1], was used for multiple comparisons in case of factors with more than two levels (P<0.05).
All statistical analyses and graphical representations were carried out with IBM SPSS Statistics (ver. 26).

1.2.Results

Among the tested EOs white thyme solubilized with the plant-based product exerted the highest insecticidal activity on green peach aphids (Tab. S1, Fig. S1), with the commercial product Prev-Am showing similar mortality (Fig. S2). Overall, zeolite decreased the EO insecticidal activity in comparison to the plant-based solubilizer (Fig. S1).

Table S1. Results of the factorial ANOVA testing the effect of EOs, solubilizers and their interaction on aphid mortality.

**Variable**	**df**	**F**	***P***
EO type	3, 102	5.09	<0.001
Solubilizer type	1, 102	17.15	<0.001
EO × Solubilizer interaction	3, 102	1.41	0.25

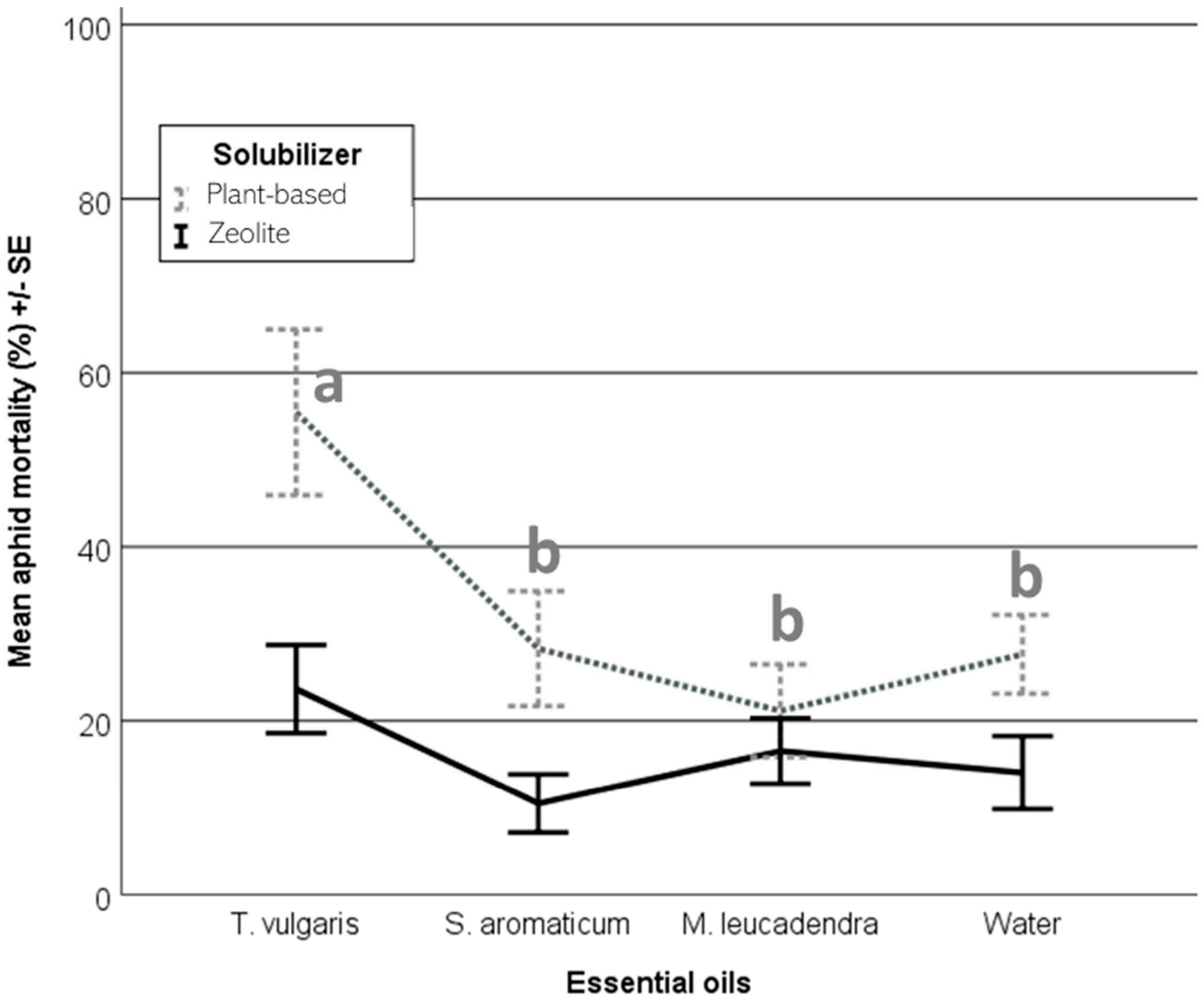

Figure S1. Effects of the different combinations of EOs and solubilizers on aphid mortality. Different letters indicate statistically significant differences according to Gabriel's test.

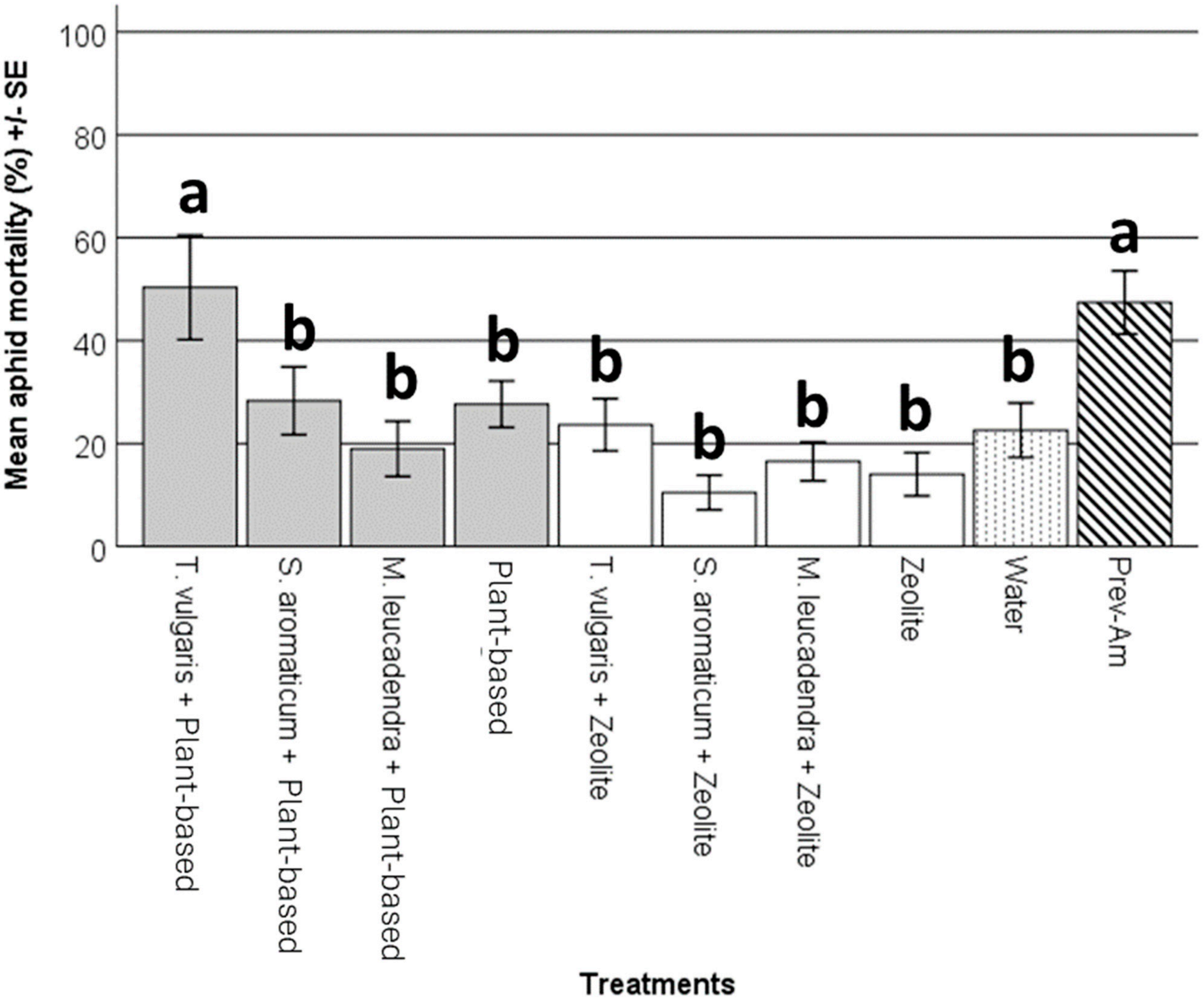

Figure S2. Effects of the different combinations of EOs and solubilizers on aphid mortality, including the commercial product Prev-Am®. One way ANOVA: $F (9, 127) = 5.05$; $P<0.001$. Different letters indicate statistically significant differences according to Gabriel's test.

Significant differences among the tested crude plant extracts emerged only if Marseille soap had been added (Tab. S2, Fig. S3). Garlic and chili pepper mixed with Marseille soap showed the highest insecticidal activity among the crude plant extract. However, no significant differences could be detected between garlic and chili pepper mixed with soap and Marseille soap alone in the water.

Based on these results, we chose white thyme solubilized with the plant-based product, Prev-Am, garlic crude extract mixed with Marseille soap and Marseille soap alone to be used in the main experiment.

Table S2. Results of the factorial ANOVA testing the effect of crude plant extracts, Marseille soap and their interaction on aphid mortality.

**Source**	**df**	**F**	***P***
Plant extract type	5, 72	2.77	0.02
Soap	1, 72	29.97	<0.001
Plant extract × Soap interaction	5, 72	5.26	<0.001

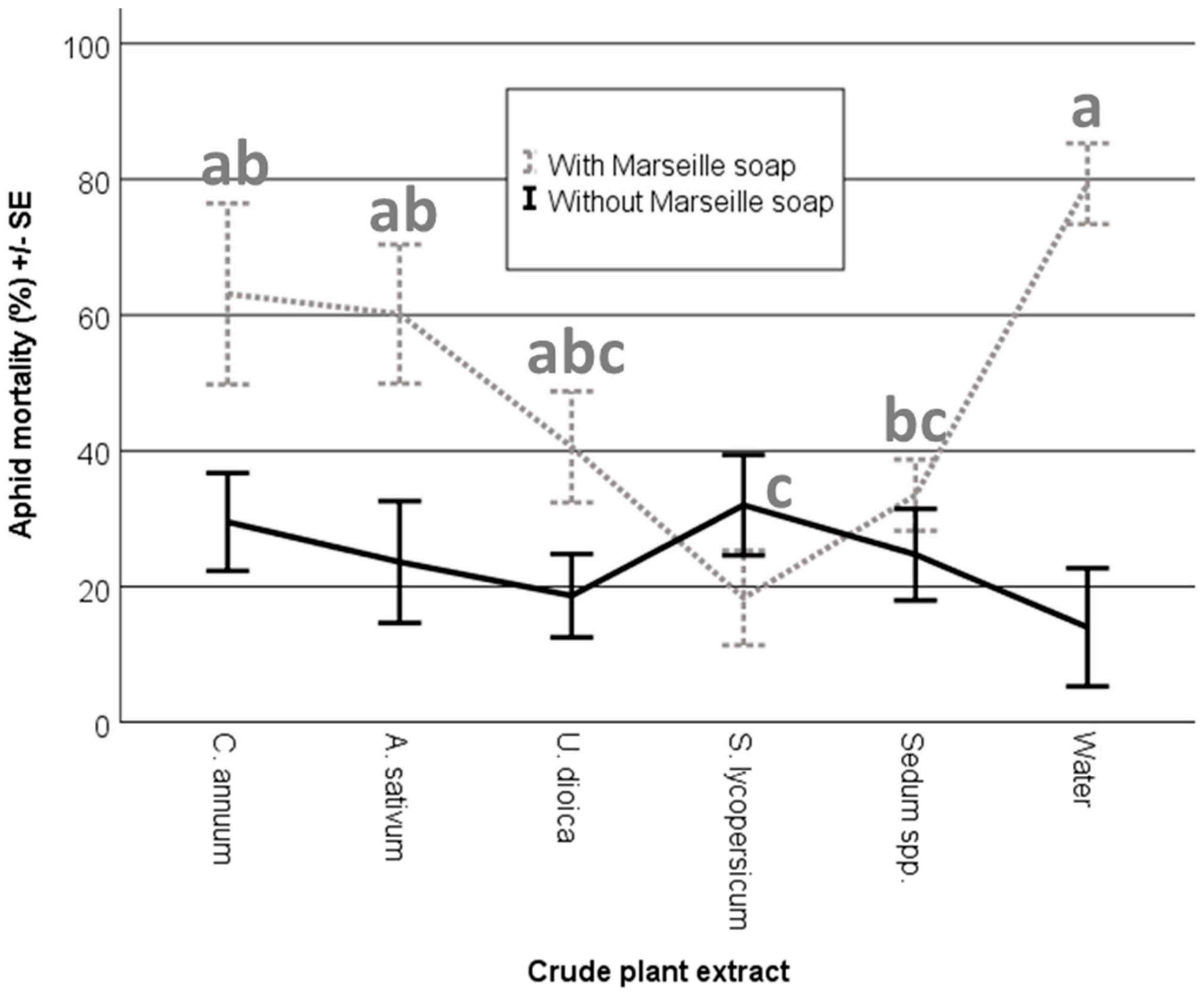

Figure S3. Effects of the different combinations of crude plant extracts and Marseille soap on aphid mortality. Different letters indicate statistically significant differences according to Gabriel's test.

2. Chemical analysis

2.1.Analysis of volatile compounds (VOCs)

VOCs analysis was performed by SPME-GC-MS according to [2], with some modifications. Approximately 2.0 ± 0.1 g of samples were weighed into a 20-mL SPME crimp neck vial. The sample was first equilibrated in the autosampler thermostat (HT2850T autosampler; HTA S.r.l., Brescia, Italy) at 40°C for 15 min; the SPME fiber (50/30 µm DVB/CAR/PDMS; Supelco Inc., Bellefonte, PA, USA) was then exposed to the headspace of the sample at 40°C for 60 min and, finally, desorbed in the injection port of the gas chromatograph-mass spectrometer (GCMS-QP2010 Plus instrument, Shimadzu Corp., Kyoto, Japan) for 30 min. VOCs were separated using a GC capillary column (Stabilwax Crossbond Carbowax polyethylene glycol; 60 mL × 0.25 mm ID × 0.25 µm film thickness; Restek Corp., Bellefonte, PA, USA). The oven temperature was held at 42°C for 5 min, then increased to 120°C at 3°C/min and to 250°C at 10°C/min (5 min hold). The injector and detector temperatures were set at 270°C and 280°C, respectively. The ion source temperature was 230°C, whereas the quadrupole mass analyzer temperature was 150°C. Helium was used as carrier gas at a flow rate of 1 mL/min. The mass spectrometer was operated by electronic impact at 70 eV, and ions were scanned over a m/z range of 33–350 at a rate of 4.43 scan/s. Mass spectra were acquired in full scan mode (total ion current); VOCs were identified by comparing their retention time and their mass spectra with those found in NIST147 library. The linear retention index (LRI) was also calculated according to the following formula:

$$KI = \left[100 * \left(tR(i) - \frac{tR(z)}{tR(z+1)} - tR(z)\right)\right] + 100z$$

where:
z is the number of carbon atoms in alkane *z*;
$tR(i)$ is the retention time of compound *i*;
$tR(z)$ is the retention time of alkane *z*;
$tR(z + 1)$ is the retention time of alkane $z + 1$.

Results were expressed in area counts, as area/sample weight (g) × 10^3. The analysis was carried out in triplicate for each sample.

2.2.Marseille soap lipid extraction

Lipids from Marseille soap were extracted according to a modified version of the Folch method [3]. About 25 g of Marseille soap were subjected to extraction by using a chloroform:methanol solution (1:1, v/v), followed by the addition of other 100 mL of chloroform. Afterwards, 1 M KCl was added, allowing the organic phase to separate. The solution was then taken to dryness and the fat content was determined gravimetrically. Three independent replicates were carried out.

2.3.Marseille soap' total fatty acids composition

To determine Marseille soap' fatty acid composition, a double methylation in methanolic medium was carried out, first with sodium methoxide and then with boron-trifluoride, to ensure that all FA (including the free ones) were completely methylated [4]. About 20 mg of sample were weighed and added with 200 µL of tridecanoic acid methyl ester (C13:0, 3.1956 mg/mL) as IS. After the addition of diethyl ether and sodium methoxide 0.5 M, the tube was placed in a boiling water bath for 20 min and cooled down to room temperature. Then, 2-3 drops of phenolphthalein 1% in methanol were added and the solution turned pink. Thereafter, boron trifluoride-methanol complex (20%) in methanol were added and the solution became colorless. The tube was put into the boiling water bath again for 15 min and cooled down to room temperature. Once the solution became colorless, n-hexane were added, the tube was shaken and added with a sodium chloride saturated solution. The two phases

were then allowed to separate, and the upper phase of n-hexane was transferred to another tube containing sodium sulphate anhydrous and was left standing for 1 h [4]. Afterwards, 1 µL of the phase containing the analytes was injected into a GC-FID GC8000 series Fisons Instruments, equipped with a split injector, and interfaced with a computerized data acquisition system (Software Chrom Card Data System ver. 2.3.1, Thermo Electron Corporation, Italy). The column used was a Restek RTX 2330 (90% biscyanopropyl, 10% cyanopropylphenyl-polysiloxane) with a length of 30 m, an internal diameter of 0.25 mm, and a film thickness of 0.2 µm [5]. Oven temperature was programmed from 100°C to 240°C at a rate of 4°C/min and the final temperature was kept for 20 min. The injector and detector temperatures were both set at 240°C. Helium was used as carrier gas at a constant pressure of 60 kPa. The split ratio was 1:30. Each fatty acid was identified by comparing its retention time with that of a commercial fatty acid methyl ester standard solution (FAME standard mix Supelco 37, Sigma, St. Louis, MO, USA). The GC response factor of each fatty acid was calculated by using the FAME standard mix and the internal standard (C13:0). The quantification of FAME was carried out according to the internal standard method. Three independent replicates were carried out.

Table S3. VOCs detected in crude garlic extract samples.

VOCs	Area	Internal distribution (%)	Linear retention index (LRI)
**Sulphur compounds**			
Allyl methyl sulfide	3821±212	0.24	956
Dimethyl disulfide	6165±128	0.38	1072
Allyl monosulfide	17145±784	1.06	1150
Propylene episulfide	7666±127	0.47	875
Methyl 2-propenyl disulfide	217941±1098	13.47	1261
Allyl disulfide	722641±2089	44.65	1463
Allyl methyl trisulfide	51920±237	3.21	1592
Di-2-propenyl trisulfide	70136±954	4.33	1819
**Total**	**1097435±10764**	**67.81**	
**Terpenes**			
Linalool	13837±854	0.86	1544
trans-citral	27976±932	1.73	1716
cis-geraniol	12899±124	0.80	1797
Nerol	102416±1112	6.33	1826
Geranyl acetate	11658±1043	0.72	1753
Caryophyllene oxide	7464±397	0.46	1980
**Total**	**176250±1873**	**10.89**	
**Alcohols**			
Ethanol	12817±365	0.79	934
3-allyl-2-methoxyphenol	37987±98	2.35	1362
**Total**	**50803±2345**	**3.14**	
**Acids**			
Hexanoic acid	8227±97	0.51	1854
Octanoic acid	136053±278	8.41	2046
Decanoic acid	108925±113	6.73	2288
**Total**	**253205±5434**	**15.65**	
**Others**			
1,2-diacetylhydrazine	40713±812	2.52	1808

Results are expressed in area counts ×10^3/g of sample for each compound and reported as mean±std dev of 3 independent replicates.

Table S4. VOCs detected in white thyme EO samples.

VOCs	Area	Internal distribution (%)	Linear retention index (LRI)
**Alkenes**			
2,7-dimethyl-3-octen-5-yne	750152±1234	2.19	2011
Tricyclo[5.3.0.0(4,8)]decane	3476420±3453	10.15	2087
9-(1-methylethylidene)bicyclo[6.1.0]nonane	1909020±98754	5.58	2121
2-pyrone, 6-pentyl	111677±1237	0.33	2175
1-isopropenyl-3-methylenecyclohexane	979477±42678	2.86	2732
7-(1-methylethylidene)bicyclo[4.1.0]heptane	1504052±189743	4.39	2890
**Total**	**8730799±2986**	**27.80**	
**Terpenes**			
trans-*p*-menthane	568636±548	1.66	1058
β-ocimene	175680±1074	0.51	1047
α-fenchene	317500±1643	0.93	1052
cis-carane	580161±987	1.69	973
dl-isopulegol	1594189±10976	4.66	1153
Thymol	8834970±48723	25.81	2189
p-cymenene	78102±194	0.23	1456
trans-linalool oxide	50180±1298	0.15	1484
Copaene	108346±3723	0.32	1470
1-methyldecahydronaphthalene	596873±1065	1.74	1489
Longifolene	205392±8512	0.60	1590
Aromandendrene	1786318±2567	5.22	1637
Epoxy-linalooloxide	67123±199	0.20	1677
Caryophyllene oxide	166885±4323	0.49	1980
1,2-dihydrolinalool	135538±8623	0.40	1509
Dihydrocarveol	483203±5129	1.41	1710
exo-fenchol	330668±7345	0.97	1785
cis-carvotanacetol	430325±3423	1.26	1800
5-caranol	1022915±12345	2.99	1886
α-terpineol	659376±5324	1.94	1563
Verbenol	22243±243	0.07	1655
trans-β-terpineol	709128±9834	2.08	1625
Neodihydrocarveol	3481718±3674	10.22	1783
p-cresol	20107±876	0.06	2087
p-toluol	58177±956	0.17	2089
p-mentha-1,8-diol	45864±234	0.13	2090
p-cymen-7-ol	6242±984	0.02	2114
6-*tert*-butyl-m-cresol	35403±1032	0.10	2260
**Total**	**22571262±75432**	**71.87**	
**Alcohols**			
5-methyl-2,4-diisopropylphenol	42723±1685	0.13	2282
2-(1,1-dimethylethyl)-5-methyl-phenol	9022±435	0.03	2310
**Total**	**51745±9323**	**0.16**	
**Acids**			
Carbonic acid, isobutyl 4-isopropylphenyl ester	53202±1003	0.17	1656

Results are expressed in area counts ×10^3/g of sample for each compound and reported as mean±std dev of 3 independent replicates.

Table S5. VOCs detected in plant-based solubilizer samples.

VOCs	Area	Internal distribution (%)	Linear retention index (LRI)
**Alkanes**			
Heptane	166055±6085	4.75	700
Nonane	231983±3498	6.64	900
4-cyclopentene-1,3-dione	35978±723	1.03	1605
Nonadecane	9898±167	0.28	1900
**Total**	**443915±5609**	**12.71**	
**Aldehydes**			
Heptanal	19070±2341	0.55	1188
Nonanal	15279±1528	0.44	1390
**Total**	**19070±1114**	**0.99**	
**Ketones**			
2-heptanone	12112±2126	0.35	1187
2-undecanone	49754±1083	1.42	1606
2-tridecanone	18215±532	0.52	1808
γ-butylbutyrolactone	6780±342	0.19	1916
5-hydroxyoctanoic acid δ-lactone	6942±189	0.20	1965
**Total**	**93803±3025**	**2.69**	
**Terpenes**			
D-limonene	8658±87	0.25	1044
p-cymen-2-ol	37308±2231	1.07	2219
**Total**	**45966±185**	**1.32**	
**Alcohol**			
1-nonanol	12971±754	0.37	1663
**Acids**			
Acetic acid	226568±1123	6.49	1480
Octanoic acid, methyl ester	125705±5489	3.60	1387
Octanoic acid, ethyl ester	19324±1083	0.55	1440
Formic acid, heptyl ester	31073±845	0.89	1528
Decanoic acid, methyl ester	57332±2984	1.64	1590
Dodecanoic acid, ethyl ester	7633±94	0.22	1856
Heptanoic acid	16984±222	0.49	1960
Octanoic acid	1582040±23679	45.29	2046
8-methylnonanoic acid	795354±1078	22.77	2050
**Total**	**2862014±3927**	**81.94**	

Results are expressed in area counts $\times 10^3$/g of sample for each compound and reported as mean±std dev of 3 independent replicates.

Table S6. VOCs detected in mix samples of thyme EO (0.25%) and solubilizer (0.5%) in water.

**VOCs**	**Area**	**Internal distribution (%)**	**Linear retention index (LRI)**
**Alkanes**			
1-isopropenyl-3-methylenecyclohexane	1684961±8432	10.83	999
1,5,5-trimethyl-4-vinyl-1-cyclopentene	279027±1923	2.79	558
9-(1-methylethylidene)bicyclo[6.1.0]nonane	841171±1171	5.41	560
1,8-dimethylspiro[4.5]decane	15431±934	0.10	570
7-(1-methylethylidene)bicyclo[4.1.0]heptane	476061±2765	3.06	602
Tricyclo[5.3.0.0(4,8)]decane	811306±5076	5.22	618
Methyl *n*-octanoate	20529±623	0.13	1387
Benzene, 1-methyl-3-(1-methylethenyl)-	50949±809	0.33	1456
**Total**	**4158905±23453**	**27.74**	
**Terpenes**			
trans-p-menthane	165049±5076	1.06	1058
p-menthane	171821±1123	1.10	1059
Ocimene	55026±3456	0.35	1047
Sabinen	138149±8123	0.89	1133
trans-carane	297687±2908	1.91	973
α-fenchene	830263±4467	5.34	1052
Myrcene	146129±5323	0.94	1159
Isopulegol	828753±7013	5.33	1153
Terpinolene	524172±9034	3.37	1280
Fenchone	15173±123	0.10	1396
Copaene	114188±5437	0.73	1470
1,3,8-*p*-menthatriene	297214±7065	1.91	1286
1,2-dihydrolinalool	71832±427	0.46	1509
Longifolene	208993±2265	1.34	1590
Exo-fenchol	158560±5234	1.02	1785
p-cymen-8-ol	30411±9876	0.20	1852
Thymol	2256215±2765	15.51	1290
2-methyldecahydronaphthalene	400415±234	2.57	1301
β-selinene	1757421±7063	11.30	1729
5-caranol	598909±9543	3.85	1886
4-caranol	237919±9234	1.53	1286
Δ-terpineol	377007±1307	2.42	1563
Verbenol	68081±871	0.44	1655
cis-verbenol	16244±576	0.10	1244
Neodihydrocarveol	223050±1034	1.43	1783
Dihydrocarveol	246280±2843	1.58	1710
Caryophyllene oxide	51857±1123	0.33	1980
Desulphosinigrin	747170±8764	4.80	1999
**Total**	**10904628±93432**	**71.12**	
**Acids**			
n-decanoic acid	111733±9324	1.14	2288

Results are expressed in area counts $\times 10^3$/g of sample for each compound and reported as mean±std dev of 3 independent replicates.

Table S7. VOCs detected in Marseille soap samples.

VOCs	Area	Internal distribution (%)	Linear retention index (LRI)
**Alkenes**			
2,4-dimethylheptane	9481±987	8.10	797
4-methyloctane	11349±568	4.74	823
2,4,6-trimethyloctane	46534±1986	4.48	1058
Undecane	6095±965	18.35	1100
2,5-dimethylnonane	11604±234	2.40	1059
4,5-dimethylundecane	9888±127	4.58	1212
4,6-dimethyldodecane	3673±98	3.90	1325
Hexadecane	2168±543	1.45	1600
4-ethylheptane	2675±835	0.86	858
4-methyldecane	1346±87	1.06	1054
2-methyldecane	9611±57	0.53	1057
Hexadecane	1488±61	0.59	1600
Undecane	1526±125	0.60	1100
Dodecane	893±12	6.61	1200
1,3-di-*tert*-butylbenzene	47545±1985	18.75	1420
**Total**	**192671±1765**	**76.00**	
**Aldehydes**			
Hexanal	9611±234	3.79	1097
Heptanal	2089±54	0.82	1188
Octanal	6274±127	2.47	1292
Nonanal	13274±345	5.23	1390
**Total**	**31228±245**	**12.32**	
**Ketones**			
2-heptanone	893±12	0.35	1187
2-nonanone	1319±76	0.52	1394
3-octen-2-one	2446±123	0.96	1411
2-decanone	1875±94	0.74	1484
**Total**	**6534±111**	**2.58**	
**Terpenes**			
D-limonene	7369±168	2.91	1044
**Alcohols**			
2-ethyl-1-hexanol	5028±273	1.98	1484
1-octanol	2311±427	0.91	1565
**Total**	**7339±98**	**2.89**	
**Acids**			
Octanoic acid	8384±638	3.31	2046

Results are expressed in area counts $\times 10^3$/g of sample for each compound and reported as mean±std dev of 3 independent replicates.

Table S8. Fatty acid and fatty acid classes (expressed as % of total fatty acids) of Marseille soap.

**Fatty acid**	**% Total Fatty Acids**
Lauric acid (C12:0)	11.63±0.61
Myristic acid (C14:0)	2.68±0.10
Palmitic acid (C16:0)	18.77±1.17
Stearic acid (C18:0)	1.47±0.29
Palmitoleic acid (C16:1)	0.20±0.00
Oleic acid (C18:1 *cis* 9)	52.66±2.18
Linoleic acid (C18:2 *cis* 9,12)	12.60±0.55
ΣSFA	34.55±2.63
ΣMUFA	52.86±2.20
ΣPUFA	12.60±0.55

Data are reported as mean±std dev of 3 independent replicates. MUFA, monounsaturated fatty acids; PUFA, polyunsaturated fatty acids; SFA, saturated fatty acids.

References

1. Gabriel, K.R. A Simple Method of Multiple Comparisons of Means. *J Am Stat Assoc* **1978**, *73*, 724, doi:10.2307/2286265.
2. Tura, M.; Ansorena, D.; Astiasarán, I.; Mandrioli, M.; Toschi, T.G. Evaluation of Hemp Seed Oils Stability under Accelerated Storage Test. *Antioxidants* **2022**, *11*, 490, doi:10.3390/antiox11030490.
3. Boselli, E.; Velazco, V.; Caboni, M.F.; Lercker, G. *Pressurized Liquid Extraction of Lipids for the Determination of Oxysterols in Egg-Containing Food*; 2001; Vol. 917;.
4. Varona, E.; Tres, A.; Rafecas, M.; Vichi, S.; Barroeta, A.C.; Guardiola, F. Methods to Determine the Quality of Acid Oils and Fatty Acid Distillates Used in Animal Feeding. *MethodsX* **2021**, *8*, doi:10.1016/j.mex.2021.101334.
5. Cardenia, V.; Rodriguez-Estrada, M.T.; Baldacci, E.; Lercker, G. Health-Related Lipids Components of Sardine Muscle as Affected by Photooxidation. *Food and Chemical Toxicology* **2013**, *57*, 32–38, doi:10.1016/j.fct.2013.02.053.
